# Polyandrous females but not monogamous females vary in reproductive ageing patterns in the bean bug *Riptortus pedestris*

**DOI:** 10.1186/s12862-022-02070-1

**Published:** 2022-10-10

**Authors:** Yi Hang Park, Donggyun Shin, Chang S. Han

**Affiliations:** Department of Biology, Kyung Hee University, Seoul, Korea

**Keywords:** Ageing, Monogamy, Polyandry, Life history

## Abstract

**Background:**

In general, reproductive performance exhibits nonlinear changes with age. Specifically, reproductive performance increases early in life, reaches a peak, and then declines later in life. Reproductive ageing patterns can also differ among individuals if they are influenced by individual-specific strategies of resource allocation between early-life reproduction and maintenance. In addition, the social environment, such as the number of available mates, can influence individual-specific resource allocation strategies and consequently alter the extent of individual differences in reproductive ageing patterns. That is, females that interact with more partners are expected to vary their copulation frequency, adopt a more flexible reproductive strategy and exhibit greater individual differences in reproductive ageing patterns.

**Methods:**

In this study, we evaluated the effect of mating with multiple males on both group- and individual-level reproductive ageing patterns in females of the bean bug *Riptortus pedestris* by ensuring that females experienced monogamous (one female with one male) or polyandrous conditions (one female with two males).

**Results:**

We found that group-level reproductive ageing patterns did not differ between monogamy-treatment and polyandry-treatment females. However, polyandry-treatment females exhibited among-individual variation in reproductive ageing patterns, while monogamy-treatment females did not.

**Conclusion:**

Our findings provide the first empirical evidence regarding the influence of the social environment on individual variation in reproductive ageing patterns. We further suggest that the number of potential mates influences group- and individual-level reproductive ageing patterns, depending on which sex controls mating. We encourage future studies to consider interactions between species-specific mating systems and the social environment when evaluating group- and individual-level reproductive ageing patterns.

## Background

A senescent decline in reproductive performance has been documented across many animal taxa [1–3]. In insects, older males are less likely to exhibit high courtship rates or frequent mating attempts, while older females display reduced oviposition rates [4]. This reproductive ageing in insects might simply be due to somatic deterioration with ageing. However, reproductive ageing can also be explained by the disposable soma theory which suggests that individuals allocate limited resources between reproduction and the maintenance of their soma [5, 6]. As a result, the nonlinear pattern of reproductive ageing can vary depending on the trade-off between allocating resources to early-life reproduction and body maintenance [7–9]. For example, high investment in early-life reproduction causes a steep increase and a peak in reproductive performance, followed by a sharp decline; individuals consequently exhibit a shortened lifespan (i.e., strongly negative quadratic reproductive ageing) due to the high energetic costs of early-life egg production [10, 11] and high rates of injury or infections obtained from multiple copulations [12, 13]. At the other extreme, low or moderate levels of reproductive investment in early life result in a gradual (rather than steep) increase in reproductive performance, a peak, and then a plateau (i.e., weakly negative quadratic reproductive ageing), resulting in a relatively long lifespan. Moreover, as nonlinear reproductive ageing patterns are determined by resource allocation strategies, they vary depending on environmental conditions that affect investment in early-life reproduction [14–16], such as food availability, population density and weather conditions (reviewed in [15]). However, previous studies concerning the effects of environmental factors on reproductive ageing patterns have focused on linear rather than nonlinear patterns [15].

In particular, the number of available male mating partners can be a potential determinant of nonlinear reproductive ageing patterns in females. Reproductive ageing patterns are expected to differ between females allowed to mate with multiple males (polyandrous females) and females that mate with only one male throughout their lifetime (monogamous females). Females can increase longevity and egg production rates by mating with multiple partners if they receive direct benefits from males during mating, such as nuptial gifts or nutritious ejaculates [17–19], and if these benefits offset the costs of reproduction. However, if males do not provide such direct benefits to females, polyandrous females are expected to experience accelerated reproductive ageing compared to monogamous females. Polyandrous females may invest more in early-life reproduction because of frequent mating with multiple males [9, 20]. Compared to monogamous conditions, in polyandrous conditions, females may also suffer from increased sexual harassment. Thus increases in both early-life reproductive performance and costs under polyandrous conditions can reduce the lifespans of polyandrous females more sharply than those of monogamous females. Alternatively, differences in reproductive ageing patterns between polyandrous and monogamous females might occur due to selection. When early-life reproductive costs are higher in polyandrous females than in monogamous females, the loss of females with high early-life reproductive output (selective disappearance, e.g., [21]) can be greater among polyandrous females than monogamous females. Therefore, both mechanisms (increased reproductive costs under polyandrous conditions and selective disappearance) predict that polyandrous females have more negative quadratic reproductive ageing curves than monogamous females.

In addition, differences in nonlinear reproductive ageing patterns between monogamous and polyandrous females in insect species are expected to depend on which sex controls mating. In insect species where females are coerced into copulation and males control mating, females are more likely to concede into superfluous copulations as the number of males increases [22–24]. These superfluous copulations result in higher costs of early-life reproduction for polyandrous females than for monogamous females, leading to faster reproductive ageing. In contrast, in insect species where males cannot coerce females to mate but instead must court them, differences in reproductive ageing patterns between monogamous and polyandrous females are expected to be less pronounced or nonsignificant. Because females control mating in such species, female copulation frequency might not increase as a function of the number of potential mates (reviewed in [24]). Thus, in such species, both polyandrous and monogamous females experience similar reproductive costs and exhibit equivalent patterns of reproductive ageing. However, even if females control mating, females under polyandrous conditions may experience increased male mating attempts and harassment, and thus incur higher reproductive costs, which could lead to faster reproductive ageing compared to monogamous females.

Moreover, female individuals are expected to vary in nonlinear reproductive ageing patterns, and the extent of individual differences in nonlinear reproductive ageing patterns within monogamous females may differ from that within polyandrous females; such differences are also expected to depend on which sex controls mating. Individuals are expected to differ in life-history strategies according to resource allocation to early-life reproduction or body maintenance [25–27], potentially generating the among-individual variation in nonlinear reproductive ageing patterns. Because individual (and genetic) variation in a trait is the raw material on which natural selection acts, the extent of individual differences in reproductive ageing patterns implies the evolvability of life-history strategies. Moreover, as genetic variation in a trait likely depends on environmental conditions (e.g., genetic variance can increase in stressful environments due to cryptic genetic variation [28]), the extent of individual variation in reproductive ageing patterns can also increase or decrease according to environmental conditions, implying that environmental conditions influence the evolution of life-history strategies. For example, in insect species where males exhibit coercive mating, all females appear to experience high rates of male harassment and superfluous copulations when males are in excess (polyandrous condition). As a result, in polyandrous conditions, coercive males are predicted to force all females to increase their early-life reproductive investment. Consequently, in insect species in which males coerce females to mate, individual differences in reproductive ageing patterns among polyandrous females are expected to be smaller than those among monogamous females. In contrast, the opposite is expected in insect species where females can reject male mating attempts and control mating. In such species, excess males provide readily available mating opportunities for females. As a result, in polyandrous conditions, some females may increase early-life reproductive investment and senesce faster, whereas others may maintain a moderate reproductive rate and focus on mating with high-quality males throughout their lifetime. Thus, in insect species where females control mating, female individuals in polyandrous conditions are expected to adopt more diverse reproductive strategies and exhibit greater individual variation in the age at peak reproductive investment, resulting in larger individual differences in reproductive ageing patterns compared to female individuals in monogamous conditions. Taken together, the species-specific mating system is predicted to influence the impact of social conditions (e.g., the number of potential mates) on the extent of individual variation in reproductive ageing patterns. However, to date, evidence of individual variation in nonlinear reproductive ageing patterns is lacking [29–31], and little is known about the relationship among mating systems, mate availability and individual variation in reproductive ageing patterns.

Here, we investigated differences in nonlinear reproductive ageing patterns between monogamous and polyandrous female bean bugs (*Riptortus pedestris*) at the group and individual levels. The bean bug *R. pedestris* (Hemiptera: Alydidae), a major pest of leguminous crops in East Asia, is a multivoltine species that produces two to three generations per year [32, 33]. Females become sexually mature within two weeks of eclosion and start to oviposit [34]; virgin females can lay unfertilised eggs throughout their lifetime [31]. Although previous research showed that mated females do not copulate for an average of 15 to 20 days after their first mating [35], our current study revealed that females mate with males every two to three days. Females of this species are known to lay eggs throughout their lifetime and to exhibit a negative quadratic effect of age on reproductive performances [31].

We conducted two independent experiments to determine which sex of *R. pedestris* controlled mating decisions (Experiment 1) and to investigate how group- and individual-level reproductive ageing patterns in females changed in response to mate availability (Experiment 2). In Experiment 1, we placed one female with a single male (and with another male prevented from accessing the female, “monogamy” treatment) or one female with two males (“polyandry” treatment), monitored mating status every 10 min for a week and then compared the copulation frequency and duration of the focal male and female between mating contexts. In Experiment 2, we reared individual females isolated from males (“no mating” treatment), housed with a single male (and with another male prevented from accessing the female, “monogamy” treatment) or housed with two males (“polyandry” treatment) and recorded their weekly egg production and lifespan. We predicted that females controlled copulation frequency and duration in *R. pedestris* because *R. pedestris* males are known to exhibit very obvious courtship behaviours [36]. Consequently, we predicted that individual variation in reproductive ageing patterns would be greater in polyandry-treatment females than in monogamy-treatment females.

## Materials and methods

### Rearing conditions

Our laboratory stock populations of *R. pedestris* were established in 2018 with hundreds of eggs that originated from a population maintained at the Pest Management Institute, Baekam, Gyeonggido, Korea. In each generation, stock populations were maintained in multiple (2 or 3) transparent plastic containers (20 × 20 × 40 cm), each containing an average of 150 adult individuals and provided with water and food (soybeans, *Glycine max* and chickpeas, *Cicer arietinum*) *ad libitum*. To generate future generations, eggs were collected on a piece of cotton placed in the containers 3 weeks after the first observation of eclosion to adulthood. We collected eggs every week, pooled them to reduce the effect of rearing containers and randomly distributed them to new containers for future generations. Stock populations and all experimental individuals were maintained at 24–27 ℃ with 40–60% relative humidity under a 17 h light:7 h dark (light: 07:00 ~ 24:00, dark: 00:00 ~ 07:00) photoperiod.

### Experiment 1. Male and female mating patterns under monogamy vs. polyandry treatments

**Fig. 1:**
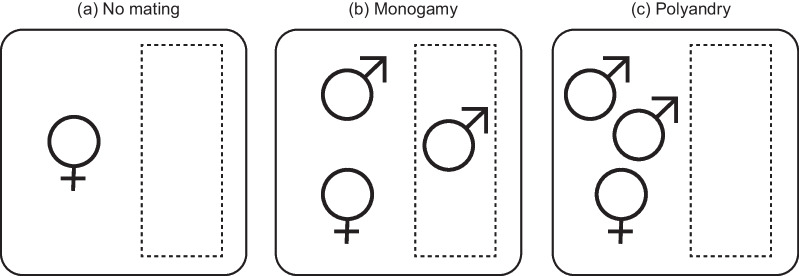
Illustration of the three mating treatments. The mating treatments manipulated the number of available mates per female. **a** No mating treatment: one female and no males; **b** monogamy treatment: one female and two males (only one male available for copulation); **c** polyandry treatment: one female and two males (both available for copulation). The small rectangular box in the home container depicts the perforated container used to physically isolate the additional male in the monogamy treatment (in both no mating and polyandry treatments, these containers were empty). Experiment 1 employed only two treatments (monogamy vs. polyandry), whereas Experiment 2 employed all three treatments

We collected newly eclosed adults from the stock populations and separated the males and females into individual home containers (females: 12 × 12 × 2 cm [width × depth × height]; males: 12 × 8 cm [diameter × height]). We kept adults in individual containers for two weeks after eclosion to ensure sexual maturation and provided food and water *ad libitum*. After two weeks, we randomly assigned sexually mature males to female home containers, establishing two distinct mating treatments: monogamy (n = 13) and polyandry (n = 19) (Fig. 1). In the “monogamy” treatment, we placed both an age-matched male (available for copulation) and another male isolated in a perforated, transparent, 10-ml conical tube with water and food (not available for copulation) in with a female. In this treatment, females could observe and smell isolated males through the tube but could not mate through it. In the “polyandry” treatment, we placed two age-matched males in with the female; thus both males were available for copulation. This design ensured that the sex ratio was held constant in both the monogamy and polyandry treatments to avoid any changes in female mating behaviour due to differences in the sex ratio [37–42]. To ensure that the environments were comparable among treatments, we also placed an empty conical tube in the home containers of polyandry-treatment females (Fig. 1). Because *R. pedestris* mating pairs display a clear end-to-end position and their copulation lasts approximately 60 to 240 min [36, 43], we were able to record the mating status of each female by photographing the home containers every 10 min. We also recorded female mating status during the dark period (00:00–07:00) by taking photographs under red LED illumination (LED T5; Zhongshan Kinsung Electronic, Zhongsan, China). One week before the experiment, we marked each male’s pronotum and abdominal sternum with enamel paint, allowing us to determine male identity in the polyandry treatment. Thus, we estimated (1) total copulation frequency and (2) total copulation duration over a week for both sexes. If a female died during the experiment (1 case in the polyandry treatment) or a male was not interested in mating (2 cases in the polyandry treatment), we excluded these data from further analysis.

### Experiment 2. Female reproductive ageing under three different mating treatments

For this experiment, we collected newly eclosed females from stock populations and placed them in individual home containers (12 × 8 cm [diameter × height]). Females were randomly assigned to one of three mating treatments (Fig. 1): no mating (44 females), monogamy (42 females) and polyandry (44 females). Females in the no mating treatment were isolated throughout their lifetime and not housed with a male. Since virgin *R. pedestris* females can lay unfertilised eggs throughout their lifespan [31], we could estimate the baseline patterns of female reproductive ageing in the absence of mating costs by measuring the production of unfertilised eggs by virgin females. In the monogamy treatment, we placed an age-matched male in with the female, allowing copulation, and physically isolated another male in a perforated transparent plastic cup (4 × 6 × 6 cm [bottom diameter × height × top diameter]), similar to the treatment in Experiment 1 (Fig. 1). In the polyandry treatment, we placed two age-matched males in with the female, allowing the female to copulate freely with either male. Empty plastic cups were also placed in the home containers of the no mating and polyandry treatments to ensure comparable environments across all treatments (Fig. 1). Water and food were provided *ad libitum* in all home containers, and a small piece of cotton paper (2 × 2 cm) was placed in each container to induce oviposition. We counted the number of eggs laid by each female and weighed each female weekly while replacing the water and food. We also checked individual survival each day to assess longevity. If a male died, he was replaced with an age-matched male from the stock population. Thus, we estimated each female’s (1) longevity, (2) lifetime egg production and (3) weekly egg production.

### Statistical analyses

#### Experiment 1

We used Mann–Whitney *U* tests to assess differences in the total copulation frequency and duration of males and females between the monogamy and polyandry treatments. When analysing the polyandry treatment data, we randomly assigned one of the two males as the “focal” male. The analysis was performed using Statistica software (version 14, TIBCO Software Inc.).

#### Experiment 2

We first analysed the effect of mating treatment on group-level reproductive ageing patterns (i.e., a linear or quadratic change in weekly egg production) using a univariate mixed-effects model, where weekly egg production was fitted as the response variable. In the model, we included female identity as a random effect and fitted mating treatment (a three-level factor: no mating, monogamy and polyandry), linear and quadratic ages (weeks since eclosion as adults), the age (in weeks) at the last observed reproduction and the interaction between mating treatment and other covariates (linear age, quadratic age and the age at the last observed reproduction) as fixed effects. In the model, we fitted age (in weeks) as age-1 (in weeks) to ensure that the intercepts of our models represented the number of eggs produced during the first week after eclosion as an adult. The age at the last observed reproduction was used to control selective disappearance [21]. In addition, because females did not differ in age at first egg production, we did not include the age at the first observed reproduction in the model.

We also constructed treatment-specific random intercept and slope models by adding random effects sequentially to assess among-individual variation in the linear/quadratic patterns of reproductive ageing in each treatment. In all the treatment-specific models below, we fitted weekly egg production as the response variable and included linear and quadratic ages (weeks since eclosion as adults) and the age (in weeks) at the last observed reproduction as fixed effects. We did not use within-individual mean-centring but rather chronological ages (in weeks) because within-individual mean-centring is sometimes problematic when calculating individual differences in ageing slopes [44, 45]. We started by fitting a treatment-specific univariate general linear model that did not include random effects, only fixed effects and residuals (Model 1). We then fitted a random intercept model that included female identity as a random factor to assess individual differences in the egg production rate (I effect, Model 2). We further fitted random slope models that sequentially included the random interaction of individual identity and linear age (I × AGE effect, Model 3) or quadratic age (I × AGE^2^ effect, Model 4) to assess among-individual variation in reproductive ageing patterns. In all the models above, residual variances were assumed to be age-specific (17 age-specific residual variances were estimated) and not correlated across ages.

All models were implemented in ASReml (version 4.2, VSN Interaction, Hemel Hempstead, UK) and solved using the restricted maximum likelihood method. We assessed the significance of fixed effects using conditional Wald F tests. The significance of variance attributable to random effects was determined using likelihood-ratio tests (LRTs) and calculated as twice the difference in log likelihood between the full model and a reduced model where the random effect of interest was removed; the P value was calculated assuming an equal mixture of P(χ^2^, df = 0) and P(χ^2^, df = 1) [46, 47]. This is indicated as “χ^2^_0/1_” in the results.

In addition to comparing models that assessed individual differences in reproductive ageing patterns, we conducted an analysis of variance (ANOVA) to compare total lifetime egg production among the mating treatments. ANOVAs were performed using Statistica software (version 14, TIBCO Software Inc.).

## Results

### Experiment 1. Male and female mating patterns under monogamy vs. polyandry treatments

**Fig. 2:**
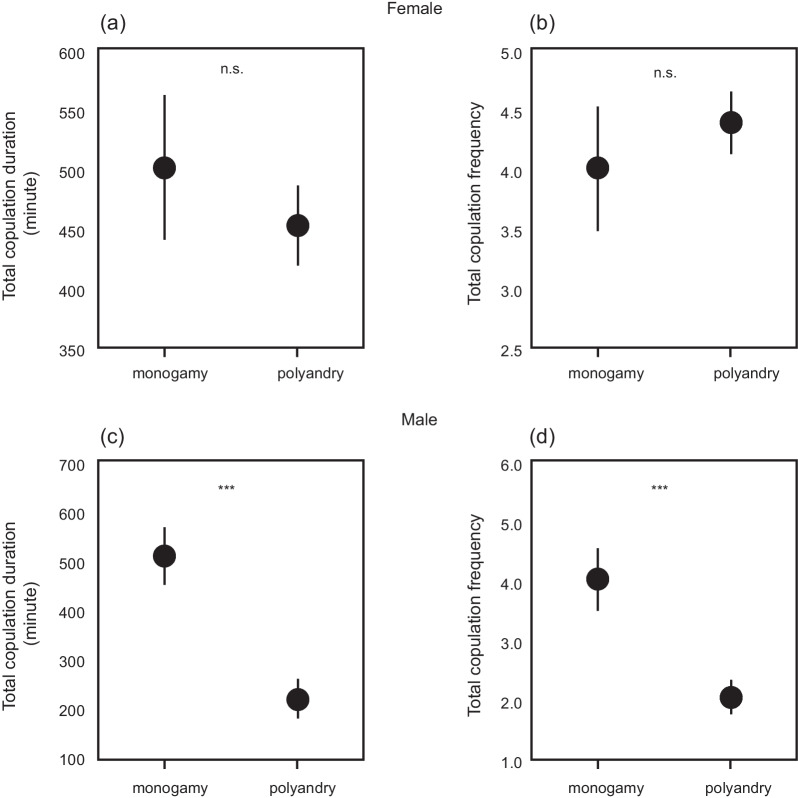
Total copulation duration **a**, **c** and frequency **b**, **d** during one week of females and males in the monogamy (n = 13) and polyandry (n = 19) treatments. ***P ≤ 0.001; n.s., P > 0.05. The dots indicate the mean values, and error bars represent standard errors of the mean

The total copulation frequency and duration of females did not differ between the monogamy and polyandry treatments (total copulation frequency: U = 92.5, Z = 1.21, *P* = 0.22; total copulation duration: U = 117.5, Z=− 0.21, *P* = 0.83) (Fig. 2). In contrast, males in the monogamy treatment had a total copulation frequency (U = 43.0, Z=− 3.06, P = 0.002) and duration (U = 29.5, Z=-3.59, P < 0.001) approximately double those of males in the polyandry treatment (Fig. 2).

### Experiment 2. Female reproductive ageing under three different mating treatments

**Fig. 3:**
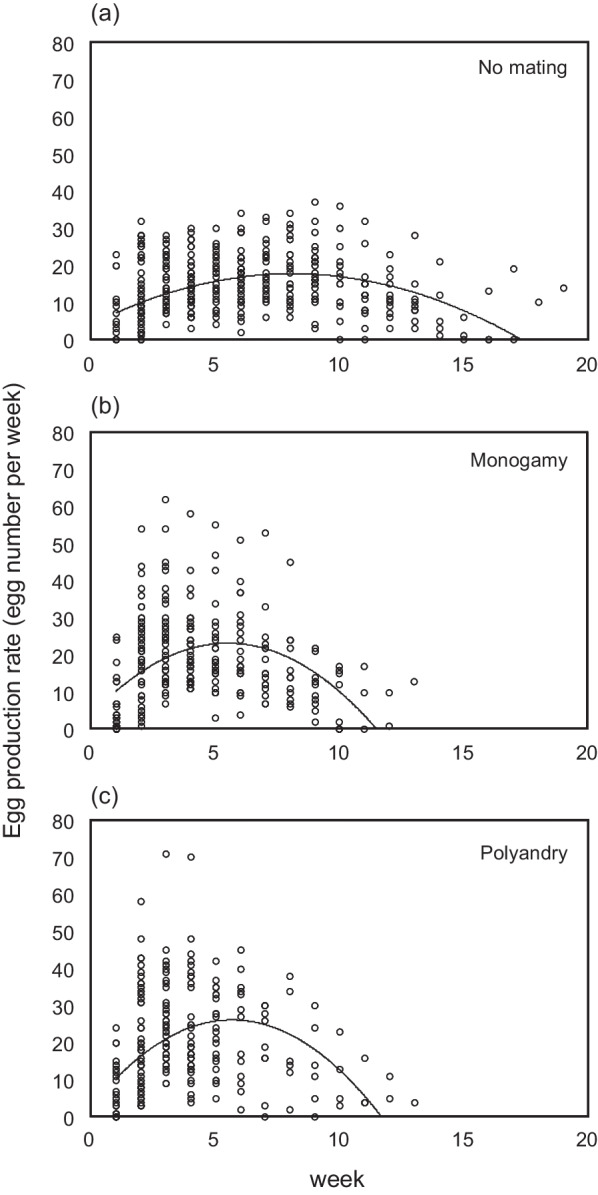
Group-level patterns of female reproductive ageing (age at egg production) under three treatments: **a** no mating, **b** monogamy and **c** polyandry

At the group level, the weekly egg production of females showed negative quadratic changes with age in all mating treatments; however, the slopes differed by treatment (Treatment × Age^2^ effect in Table 1; Fig. 3). The egg production of mated females in the monogamy and polyandry treatments peaked earlier, increased more sharply and decreased more sharply around the peak than that of virgin females (Treatment × Age^2^ effect in Table 1; Fig. 3). However, the negative quadratic change in weekly egg production did not differ between the monogamy and polyandry treatments (Fig. 3). Additionally, selective disappearance did not influence age-related changes in weekly egg production (Table 1). The total number of eggs produced did not differ among mating treatments (ANOVA, df = 2, F = 1.04, P = 0.35). In addition, mated females (monogamy- and polyandry-treatment females) tended to be heavier than virgin females (F_2,126.8_=2.58, P = 0.08), possibly due to the production of fertilised eggs. However, there was no difference in female body weight between the monogamy and polyandry treatments (F_1,84.0_=0.01, P = 0.92).

**Table 1:** Effects of mating conditions (no mating, monogamy and polyandry treatments) on group-level reproductive ageing patterns (age dependency of female egg production)

Fixed effect	β (s.e.)	F_NUMdf,DENdf_	P
Intercept	3.74 (3.06)	6.29_1,287.6_	0.01
Age	3.28 (0.36)	199.22_1,71.2_	<0.001
Age^2^	− 0.22 (0.02)	237.44_1,13.1_	<0.001
Treatment		6.47_2,120.6_	0.002
Monogamy	1.91 (4.80)		
Polyandry	1.70 (4.50)		
Treatment × age		12.28_2,391.3_	<0.001
Monogamy × age	3.76 (0.82)		
Polyandry × age	2.35 (0.87)		
Treatment × age^2^		36.51_2,289.0_	<0.001
Monogamy × age^2^	− 0.48 (0.07)		
Polyandry × age^2^	− 0.40 (0.07)		
Last reproduction^a^	0.18 (0.31)	1.38_1,127.7_	0.24
Treatment × last reproduction		0.82_2,128.5_	0.44
Monogamy × last reproduction	− 0.20 (0.58)		
Polyandry × last reproduction	0.65 (0.60)		

^a^Last observed week of reproduction

**Fig. 4:**
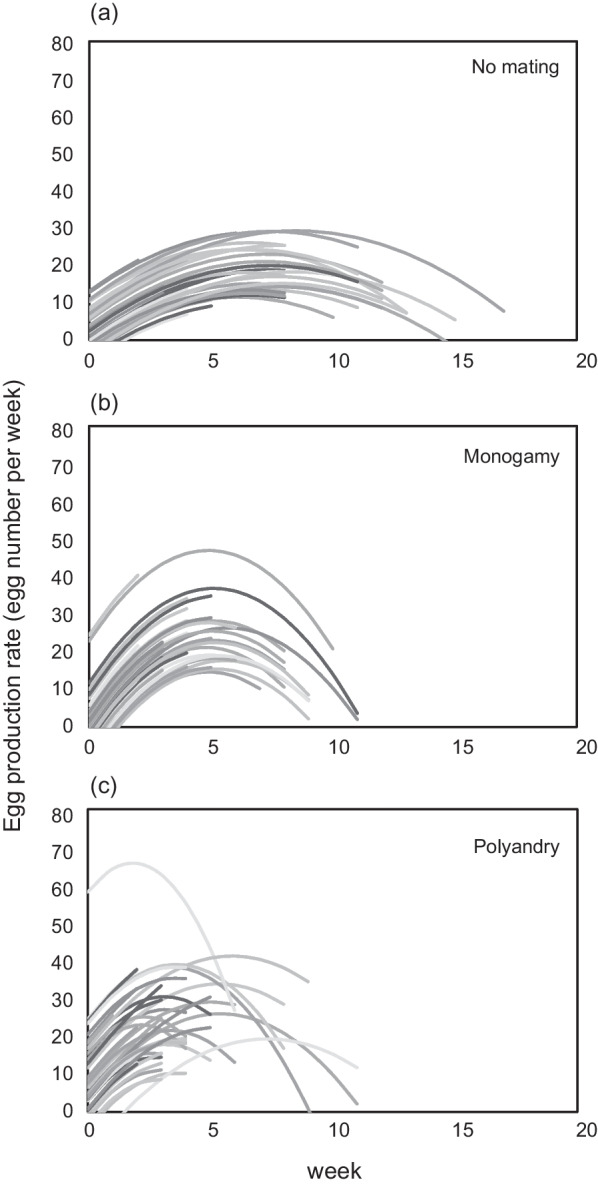
Individual-level patterns of female reproductive ageing (age at egg production) under the three treatments: **a** no mating, **b** monogamy and **c** polyandry. Lines indicate different individuals. Individual-specific slopes and intercepts were based on the best linear unbiased predictors

At the individual level, in all mating treatments, there were strong differences in egg production (I effect, Table 2). Individuals in the no mating or polyandry treatments varied in their linear age-related change in weekly egg production, whereas individuals in the monogamy treatment did not vary (I × AGE effect, Table 2). The negative quadratic change in weekly egg production did not differ among individuals in the no mating or monogamy treatments (Table 2; Fig. 4) but differed among individuals in the polyandry treatment (I × AGE^2^ effect, Table 2; Fig. 4).

**Table 2:** Hierarchical mixed-effects models assessing individual variation in linear or nonlinear (quadratic) reproductive ageing patterns

		No mating		Monogamy		Polyandry	
Random effect	Model comparisons	χ^2^_1_	P	χ^2^_1_	P	χ^2^_1_	P
I	1 vs. 2	158.91	< 0.001	104.05	< 0.001	18.61	< 0.001
I × AGE	2 vs. 3	12.41	< 0.001	0.13	0.72	6.79	0.009
I × AGE^2^	3 vs. 4	2.20	0.14	2.59	0.11	17.95	< 0.001

Significance was based on χ^2^ values derived from likelihood-ratio tests comparing models (models 1–4), as described in the "Materials and methods" section

## Discussion

Our results show that the social environment, such as the number of potential mates, influences individual- as well as group-level patterns of reproductive ageing in females of the bean bug *R. pedestris*. Females that mated with only one male throughout their lifetime (i.e., monogamy-treatment females) did not vary in their nonlinear reproductive ageing patterns. However, when the number of potential mates increased (i.e., polyandry-treatment females), females exhibited variation in their nonlinear reproductive ageing patterns. This result was in line with our prediction because females control copulation frequency and duration in *R. pedestris*. Thus, we suggest that species-specific mating systems (e.g., which sex controls mating) and mate availability (e.g., the number of opposite-sex mating partners) can shape individual variation in reproductive ageing patterns in insects.

Mated females showed faster reproductive ageing than virgin females that laid unfertilised eggs, implying the presence of costs related to mating and the production of fertilised eggs. However, we did not find differences in lifetime egg production between monogamous and polyandrous females, indicating that their lifetime reproductive costs were similar. This idea was also supported by the lack of differences in group-level nonlinear reproductive ageing patterns between monogamous and polyandrous females. In *R. pedestris*, females control mating and copulate only when males mount females and display courtship behaviours, including up and down movements of their antennae and forelegs [36]. That is, *R. pedestris* males cannot coerce females to mate. In our observation, when females are reluctant to mate, they actively kick males with their hind legs, preventing males from mounting them and displaying courtship signals. Additionally, we found that there were no differences in copulation frequency or duration between monogamous and polyandrous females and that males in the polyandry treatment experienced copulation frequencies and durations that were half those of males in the monogamy treatment. This result indicates that *R. pedestris* females that live with multiple potential mates refuse to engage in prolonged mating with a single male. Thus, as *R. pedestris* females control mating decisions, female copulation frequency is not a function of the number of potential mates, resulting in the lack of differences in group-level nonlinear reproductive ageing patterns between females in the monogamy and polyandry treatments.

Despite the lack of differences in group-level reproductive ageing patterns between treatments, polyandry-treatment females exhibited significant among-individual variation in their nonlinear reproductive ageing patterns, while monogamy-treatment females did not. One explanation for this difference is that the increase in the number of potential mates did not affect female copulation frequency at the group-level but increased the among-individual variation in female copulation frequency. That is, females might vary in reproductive strategies (e.g., choosy versus indiscriminate mating behaviour) in response to increased mate availability [48, 49]; this variation would lead to increased individual differences in nonlinear reproductive ageing patterns as well as in life-history strategies. Theoretically, females make flexible reproductive decisions when they can assess demographic conditions (e.g., mate availability) and evaluate the quality of potential mates [48, 49]. *R. pedestris* females prefer males that have longer hind legs and produce more rapid courtship tapping [50], indicating that they assess the quality of potential mates. This preference also implies that *R. pedestris* females will vary in their copulation frequency and reproductive strategies when multiple mates are available, thus resulting in greater individual differences in reproductive ageing patterns. For example, in the polyandry treatment that provides the opportunity to mate with multiple available males, some females may increase copulation frequency with multiple males early in life (i.e., increased investment in early-life reproduction) and experience rapid reproductive ageing, whereas other females may be choosy, maintaining moderate copulation frequency throughout their lifetime and experiencing slow reproductive ageing. In contrast, in the monogamy treatment where only a single male is available, females could not be choosy. Although there was an extra male in the monogamy treatment, he was unavailable for copulation. Thus, females were not in direct contact with him, were unable to precisely assess his quality and could not be choosy. We also found that female copulation frequency did not differ between the monogamy and polyandry treatments, suggesting that monogamy-treatment females were less choosy (or not choosy) than polyandry-treatment females. Therefore, all the monogamy-treatment females indiscriminately allowed male mating attempts and showed similar negative quadratic reproductive ageing patterns among individuals.

Therefore, in insect species in which females control mating decisions, increased mate availability facilitates variation in female reproductive strategies, leading to an increase in the extent of individual differences in nonlinear reproductive ageing patterns. However, the opposite is expected in insect species in which males obtain copulations through coercion. In such species, more females are predicted to experience multiple copulations as the number of males increases, potentially decreasing individual differences in copulation frequency as well as in reproductive ageing patterns in females. Thus, future studies should determine how species-specific mating systems (e.g., which sex controls mating decisions) interact with mate availability to influence the extent of individual differences in reproductive ageing patterns as well as group-level reproductive ageing patterns.

Our results also suggest that social environment factors, such as the sex ratio, affect not only group-level female reproductive ageing patterns but also the extent of among-individual variation in female reproductive ageing patterns in insects. As the sex ratio is known to affect the cost of early-life reproduction as well as age-specific mortality [51], it may also alter reproductive ageing patterns (e.g., [52]). In conditions with a male-biased sex ratio, strong male–male competition may enhance courtship and postcopulatory guarding efforts in males [53]; however, the increased rates of male harassment and copulations may harm females [54, 55]. Thus, under a male-biased sex ratio, increased reproductive costs – for both males and females – are predicted to lead to increased investment in early-life reproduction, reduced longevity and faster reproductive ageing. For example, in the field cricket *G. campestris*, male calling efforts declined with age in groups with a balanced sex ratio but did not decline in groups with a female-biased sex ratio [52]. Moreover, the sex ratio is expected to affect the extent of among-individual variation in female reproductive ageing patterns, which might also be dependent upon which sex controls copulation. In insect species in which females control copulation, a male-biased sex ratio increases mate availability for females, enabling them to be choosy [48, 49] and vary their reproductive strategies. Thus, a male-biased sex ratio can increase individual differences in the reproductive ageing patterns of females in species where females control mating. In contrast, in insect species in which males control copulation, a male-biased sex ratio forces all females to experience high copulation frequency and thus decreases individual differences in the reproductive ageing patterns of females. For example, in the green-veined white butterfly (*Pieris napi*), males cannot coerce females to mate; thus, females exhibit among-individual variation in mating frequency even with a male-biased sex ratio [56]. This variation might lead to individual differences in life-history strategies and reproductive ageing patterns in females.

In addition to the social environment, (early or current) environmental quality is expected to influence the extent of variation in individual- and group-level patterns of reproductive ageing in insects. Environmental effects on individual or genetic variation in ageing have rarely been studied in vertebrates (but see [57]), and, to date, there have been no relevant studies on invertebrates such as insects. However, there are several predictions regarding the effects of the environment. First, individual differences in reproductive ageing patterns can be larger in high-quality environments than in low-quality environments. Individuals in high-quality environments may vary their life-history strategies according to their physiological state, which would increase individual differences in reproductive ageing patterns. In contrast, in low-quality environments, all individuals may delay reproduction, thus decreasing individual differences in reproductive ageing patterns. However, the opposite is also expected. High-quality environments may increase the reproductive ageing of all individuals (the live-fast die-young hypothesis, [58]) or decrease the reproductive ageing of all individuals (the silver spoon hypothesis, [59]), leading to similar reproductive ageing patterns among individuals. Moreover, in low-quality environments, some individuals may increase early-life reproductive investment and experience fast reproductive ageing, whereas others may delay reproduction and wait for resources to become plentiful; such discrepancies result in increases in the extent of individual differences in reproductive ageing patterns. For example, in three-spined sticklebacks (*Gasterosteus aculeatus*), the throat colour of males (red; a sexual signal) changes with age; no significant genetic variation in colouration-indicated ageing was found in males who experienced warm winters (a high-quality environment) in their juvenile stage, whereas males who experienced temperature regimes equivalent to those in the wild over the winter (a low-quality environment) exhibited genetic variation in colouration-indicated ageing [57]. In the green-veined white butterfly (*P. napi*) that we mentioned above, individual differences in female mating frequency were suggested to stem from unpredictable weather conditions [56]. These findings may imply that unfavourable environments, such as unpredictable or unfavourable weather conditions, increase individual differences in reproductive ageing patterns as well as in the timing of peak reproductive investment. A meta-analysis also found mixed results concerning the effect of environmental quality on the genetic (co)variance of traits [58]; thus, the influence of the environment on genetic or individual variance in age-related plasticity, such as reproductive ageing patterns, is also expected to be species specific. Future studies need to assess how genetic or individual variation in reproductive ageing patterns, as well as group-level reproductive ageing patterns, is influenced by environmental changes.

In the present study, we demonstrated that group-level reproductive ageing patterns in *R. pedestris* females did not change but that females exhibited individual differences in their patterns of reproductive ageing with increases in the number of potential mates. Previous studies have assessed the relationship between ageing and the social environment at the group level (reviewed in [15]). Here, we provide the first evidence that changes in the social environment (e.g., the number of potential mates) can alter the extent of individual variation in reproductive ageing patterns. Although individual variation in reproductive ageing patterns can be shaped by variation in the developmental (micro)environment of individuals (e.g., brood size) [16], we further suggest that the extent of individual variation in reproductive ageing patterns depends on current environmental conditions, which all individuals experience. As individual-level reproductive ageing patterns reflect life-history strategies, studying environmental influences on individual differences in reproductive ageing patterns can enhance our understanding of the evolution of life-history strategies in response to changing environments [60, 61]. Our results also suggest that the effect of the social environment on individual differences in reproductive ageing patterns depends on which sex controls copulation. Therefore, the next step in this line of research is to assess how species-specific mating systems (i.e., the sex that controls mating) and the social environment interact to shape group- and individual-level reproductive ageing patterns, thereby providing a better understanding of plasticity in life-history strategies in both vertebrates and invertebrates.

## Data Availability

The datasets used and/or analysed during the current study are available from the corresponding author on reasonable request.
